# The use of a prefabricated radial forearm free flap for closure of a large tracheocutaneous fistula: a case report and review of the literature

**DOI:** 10.1186/s13256-015-0728-z

**Published:** 2015-11-01

**Authors:** Allison K. Royer, Mark C. Royer, Jonathan Y. Ting, Edward C. Weisberger, Michael G. Moore

**Affiliations:** 1 Columbus ENT and Allergy, Columbus Regional Health, Columbus, IN USA; 2 Department of Otolaryngology-Head and Neck Surgery, Indiana University School of Medicine, 550 North University Boulevard, Room 3170, Indianapolis, IN 46202 USA

**Keywords:** Radial forearm free flap, Tracheocutaneous fistulae

## Abstract

**Introduction:**

The closure of complex tracheocutaneous fistulae is a surgical challenge. We describe a staged approach for management of a patient with a large tracheocutaneous fistula in the setting of prior surgery and local radiation therapy.

**Case presentation:**

A 66-year-old Caucasian man who had undergone prior surgery and radiation therapy to the lower neck presented to our hospital for treatment of a large tracheocutaneous fistula that had developed with an adjacent area of tracheal stenosis. A prefabricated composite graft made up of an inner mucosal lining (buccal mucosa), a central cartilage structure (conchal cartilage), and external skin lining was constructed on the patient’s distal volar forearm and subsequently harvested in a staged fashion. This graft was transferred as a free flap and successfully used to close the patient’s defect following revascularization. Sixty months after surgery, the patient had no airway compromise or new dysphonia.

**Conclusions:**

The use of a prefabricated mucosally lined composite graft can allow for successful closure of large tracheocutaneous fistulae, even in the setting of prior radiation therapy.

## Introduction

Tracheocutaneous fistulae (TCF) typically form as sequelae of tracheostomy tube placement. Squamous epithelium migrates into the trachea, creating an epithelialized tract that fails to close following tube removal. Factors such as treatment with radiation therapy and prolonged tracheostomy tube placement increase the likelihood of fistula formation. Problems associated with TCF include increased possibility of respiratory tract infections, difficulty with phonation, coughing, cosmetic problems, and limitations on swimming and bathing, among others.

Although management of smaller TCF is often safely and successfully accomplished by performing limited local procedures, closure of large tracts (e.g., greater than 4 mm) can lead to a greater risk of perioperative complications such as subcutaneous emphysema, pneumothorax, and respiratory compromise [1]. Moreover, when the defect involves a large component of the circumference and structural support of the anterior trachea, the adequacy of the repaired airway must be taken into consideration [2–5].

We present a case report of a successful three-staged repair of a large TCF in a patient who had undergone prior tracheal surgery and radiation therapy.

## Case presentation

**Fig. 1:**
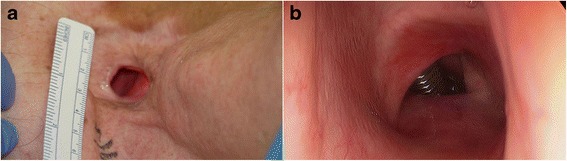
**a** The patient’s tracheocutaneous fistula defect included the entire anterior tracheal wall and a portion of the bilateral sidewalls. **b** A bronchoscopic view shows the tracheocutaneous fistula with an endotracheal tube passed through it. Proximal tracheal stenosis is evident

A 66-year-old Caucasian man presented to our hospital with a history of thyroid lymphoma treated with chemoradiation therapy (60 Gy). Subsequent surveillance revealed a new lesion within the thyroid gland, and workup demonstrated a localized papillary thyroid carcinoma treated with total thyroidectomy. Postoperatively, he developed left true vocal fold paralysis due to close adherence of the primary tumor to the nerve, and 4 months later he developed an acute episode of airway distress, presumably caused by laryngeal edema, that required an emergent tracheostomy. The laryngeal edema gradually resolved, and his tracheostomy tube was successfully downsized and he was decannulated. He was then noted to have both a small tracheoesophageal fistula as well as a large TCF. The tracheoesophageal fistula was successfully managed with a sternocleidomastoid muscle rotational flap. Bronchoscopy revealed a TCF that was 1.4 cm × 1.6 cm in size and had an adjacent 1.0-cm section of proximal tracheal stenosis (Fig. 1). Given his history of radiation, failure of previous primary and rotational graft closure, and the size and complexity of the TCF, a decision was made to repair the defect using a three-stage approach.

**Fig. 2:**
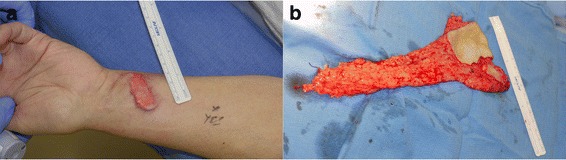
**a** The conchal cartilage covered with buccal mucosa and implanted in the radial forearm is shown before the start of stage 3. **b** The radial forearm free flap with the mucosa-lined cartilage graft and skin paddle following harvest during stage 3

The initial stage involved implanting a conchal cartilage graft with the concave portion facing superficially on the ulnar aspect of the distal left forearm. Two months later, owing to the patient’s dense hair distribution over the ulnar forearm, a second-stage procedure was performed to place a buccal mucosal graft over the conchal cartilage graft after removing the hair-bearing skin. After allowing 6 weeks for healing, the third stage was performed. This stage involved transferring the radial forearm free flap (RFFF) with cartilage and mucosal graft for closure of the TCF and augmentation of the associated tracheal stenosis (Fig. 2).

**Fig. 3:**
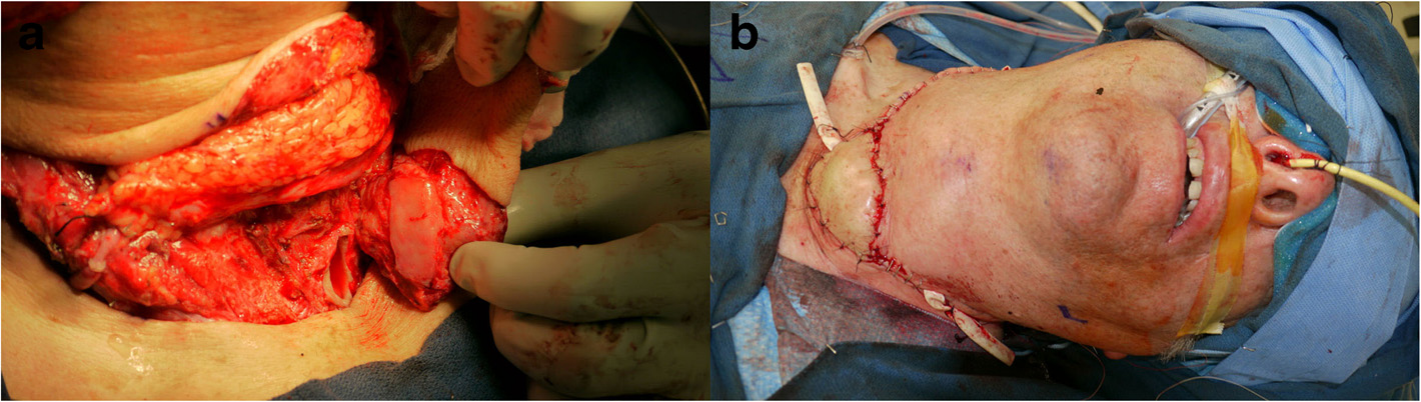
The radial forearm free flap was inset, and the radial artery and cephalic veins were anastomosed to the right superior thyroid artery and external jugular vein, respectively. **a** The mucosa-lined cartilage was positioned over the tracheal defect and secured. **b** The subcutaneous tissue and skin of the radial forearm free flap served as additional tissue support in final wound closure. Drains were placed in the neck

During this procedure, the conchal cartilage covered by mucosa was inset into the tracheal defect with interrupted and vertical mattress 2-0 polydioxanone sutures, followed by application of a layer of fibrin glue. Support was augmented with a 1.2-mm absorbable miniplate (LactoSorb; W. Lorenz Surgical, Jacksonville, FL, USA). Two of these plates were used to secure the free flap graft to a trachea (one superiorly and one inferiorly). A narrow strip of the distal flap was deepithelialized to allow for the remaining skin to be used in the external skin closure. Additional RFFF soft tissue was positioned between the tracheal closure and skin. The radial artery was anastomosed to the right superior thyroid artery, and the cephalic vein was anastomosed to the right external jugular vein. Penrose drains were placed in the neck (Fig. 3).

The patient was extubated in the operating room following reconstruction. The patient was advised to avoid straining, speaking, and coughing in the immediate postoperative period. He had no issues with breathing or wound healing in the immediate postoperative period or at 6, 12, 18, and 60 months postoperatively.

## Discussion

Although the occurrence of TCF is not uncommon in the pediatric population (11–43 % of the time following decannulation) [6–8], the literature is fairly sparse on the occurrence of TCF in adults. Although small TCF are often successfully managed with simple tract excision and/or layered closure, large TCF often require a more definitive procedure, as it is necessary not only to close the defect but also to reconstruct the missing component of the tracheal wall.

Various methods have been described for closure of large TCF. In short-segment lesions, when the healing characteristics of the wound bed are appropriate, a tracheal resection and end-to-end anastomosis or sliding tracheoplasty can be performed. When patient factors (e.g., diabetes mellitus, chronic lung disease, malnutrition, or chronic steroid use) or treatment factors (e.g., prior neck irradiation) limit wound healing, the risks of such an operation often outweigh the potential benefits. In such instances, surgeons began to look for local or regional solutions to the problem.

In 1934, Jackson and Babcock described using a lined, bipedicled flap reinforced with conchal cartilage for closure [9]. Authors of several subsequent case reports and case series have described various techniques involving different types of flaps, sometimes combined with rib cartilage for structural support [10–12]. All of these techniques had small sample sizes with variable follow-up. Whether the patients in these reports had been previously irradiated was not always discussed.

More significant tracheal replacement and reconstruction are a challenge for any surgeon. An excellent overview on the subject is provided in a review by Grillo [13], a pioneer in tracheal surgery. Belsey emphasized requirements for successful tracheal replacement, such as a repair that is laterally rigid but longitudinally flexible as well as a surface epithelium that is ciliated respiratory epithelium, if possible [14]. The lateral requirement has been shown to be desirable but not essential [15, 16]. Substances used should be biocompatible and/or inert, nontoxic, noncarcinogenic, and, if not used along with immunomodulating agents, nonimmunogenic [17, 18].

Numerous reports in recent years have described use of fasciocutaneous flaps with costal cartilage as structural support [2–4]. Although this support technique provides a large amount of rigid grafting material, its harvest includes multiple risks (such as pneumothorax, hemothorax, and injury to intercostal neurovascular bundle) as well as postoperative morbidity (chest wall pain leading to atelectasis). Moreover, its straight shape makes reconstruction of the anterior tracheal ring more challenging, unless the cartilage is fragmented. This approach is well suited for long anterior wall defects but may not be necessary for shorter repairs.

Our technique represents a novel approach in a previously irradiated patient who had undergone multiple neck surgeries before TCF closure. As a result, we needed to combine the principles of TCF repair (removal of the epithelial tract, minimizing surrounding dissection, achieving a watertight closure, and using passive drains) and tracheal repair (minimize wound tension and positive pressure across the repair in the postoperative period). In our patient, the banked mucosally lined cartilage served as a framework for structural support of the trachea, augmented with an absorbable reconstructive miniplate. The cartilage curvature allowed for reconstruction of the anterior tracheal wall in a manner more natural than would have been provided by costal cartilage. Moreover, the morbidity of the chest wall defect was avoided, allowing for easier maintenance of lung reexpansion in the postoperative period. The use of the RFFF as a grafting material provided a source of healthy, nonirradiated tissue and additional support between the tracheal closure and the skin. The use of passive drains and voice rest helped minimize stress on the tracheal anastomosis to allow for an airtight seal to be achieved.

## Conclusions

Large TCF in previously irradiated patients present a surgical challenge. This case illustrates successful closure of an extensive TCF involving the anterior trachea and partial bilateral sidewalls. The composite graft allowed for structural support with hairless mucosal lining within a fasciocutaneous flap. This surgical technique and postoperative management strategy can be considered in patients who present with similar TCFs.

## Consent

Written informed consent was obtained from the patient for publication of this case report and any accompanying images. A copy of the written consent is available for review by the Editor-in-Chief of this journal.

## Abbreviations

RFFF: Radial forearm free flap
TCF: Tracheocutaneous fistulae
